# Comparative Insights on Inpatient Outcomes in Diastolic Heart Failure with and Without Amyloidosis: A Nationwide Propensity-Matched Analysis

**DOI:** 10.3390/jcdd12050190

**Published:** 2025-05-16

**Authors:** Aravind Dilli Babu, Mirza Faris Ali Baig, David A. Baran, Jerry Estep, David Wolinsky, Nina Thakkar Rivera, Ram Bhutani, Harshit Narula, Prashant Chaulagain, David Snipelisky

**Affiliations:** 1 Department of Internal Medicine, Sinai Hospital of Baltimore, Baltimore, MD 21215, USA; rbhutani@lifebridgehealth.org (R.B.); jnarula@lifebridgehealth.org (H.N.); pchaulagain@lifebridgehealth.org (P.C.); snipeld@ccf.org (D.S.); 2 Department of Internal Medicine, Asante Three Rivers Medical Center, Grants Pass, OR 97527, USA; baigfaris90@gmail.com; 3 Advanced Heart Failure and Transplantation Section of Heart Failure & Cardiac Transplant Medicine, Cleveland Clinic, Weston, FL 33331, USA; barand@ccf.org (D.A.B.); riveran15@ccf.org (N.T.R.); 4 Department of Cardiology, Cleveland Clinic, Weston, FL 33331, USA; estepj@ccf.org (J.E.); wolinsd@ccf.org (D.W.)

**Keywords:** amyloidosis, cardiovascular disease, heart failure, propensity score match

## Abstract

Cardiac amyloidosis (CA), an infiltrative restrictive cardiomyopathy, is a frequently underrecognized etiology of diastolic heart failure (HF). This study aimed to evaluate inpatient outcomes among patients hospitalized with decompensated diastolic HF with and without a secondary diagnosis of amyloidosis, utilizing data from the National Inpatient Sample (2018–2021). Among 2,444,699 patients hospitalized for decompensated diastolic HF, 9205 (0.3%) had a documented secondary diagnosis of amyloidosis. After 1:1 propensity-score matching, 1841 patients in each group were analyzed. Multivariate logistic regression revealed that the presence of amyloidosis was associated with significantly higher odds of in-hospital mortality (4.0% vs. 2.7%), cardiogenic shock (5.4% vs. 2.4%), acute kidney injury (28.3% vs. 22.0%), ventricular tachycardia (12.4% vs. 6.0%), and acute myocardial injury (9.5% vs. 6.0%) (all *p* < 0.05). Additionally, patients with amyloidosis had a longer mean length of stay (7.1 vs. 5.7 days) and higher mean hospitalization costs ($85,594 vs. $48,484, *p* < 0.05). Although the overall incidence of acute myocardial injury was elevated, subgroup analysis of ST-elevation and non–ST-elevation myocardial infarction revealed no significant differences. These findings underscore the considerable clinical and economic burden of amyloidosis in patients hospitalized with decompensated diastolic heart failure.

## 1. Introduction

Cardiac amyloidosis (CA) presents as an infiltrative and restrictive cardiomyopathy and often leads to diastolic heart failure (HF). There are two main subtypes: transthyretin cardiac amyloidosis (ATTR-CA) and immunoglobulin light-chain cardiac amyloidosis (AL-CA). ATTR-CA, typically diagnosed among patients of older age, is predominantly found in males [1]. Studies indicate that amyloidosis is under-recognized in the population of patients with HF [2]. A meta-analysis suggested a prevalence of 13.7% [3], while autopsy studies have revealed ATTR in 25% of individuals over 80 years old [4]. When the disease is untreated, the median survival is poor: 2.5 years for hereditary ATTR (ATTRh-CA) and 3.6 years for wild-type ATTR (ATTRwt-CA) [5]. Advances in bone scintigraphy, coupled with increased awareness and availability of treatment options, have led to greater recognition of this disease [6]. Our understanding of amyloidosis prevalence and its impact on HF outcomes mainly stems from small, single-center studies, which may not fully represent the broader population [2,7]. This study used a large national sample to evaluate the inpatient outcomes for patients with decompensated diastolic HF with and without amyloidosis.

## 2. Methods

### 2.1. Study Design and Population

Data from the National Inpatient Sample (NIS) databases dated between 1 January 2018 and 31 December 2021, were utilized to analyze hospitalizations for decompensated diastolic HF. Patients were categorized based on the presence of amyloidosis and matched using propensity scores in a 1:1 ratio. The NIS, a component of the Healthcare Cost and Utilization Project (HCUP) supported by the Agency for Healthcare Research and Quality (AHRQ), stands as the largest publicly accessible nationwide database in the United States [8]. The NIS gathers patient- and hospital-level data from non-federal acute-care US hospitals and employs systematic sampling techniques, capturing 20% of discharge records from all HCUP-participating US community hospitals, except for long-term acute care facilities and rehabilitation centers. Each discharge is weighted to ensure national representativeness.

**Figure 1:**
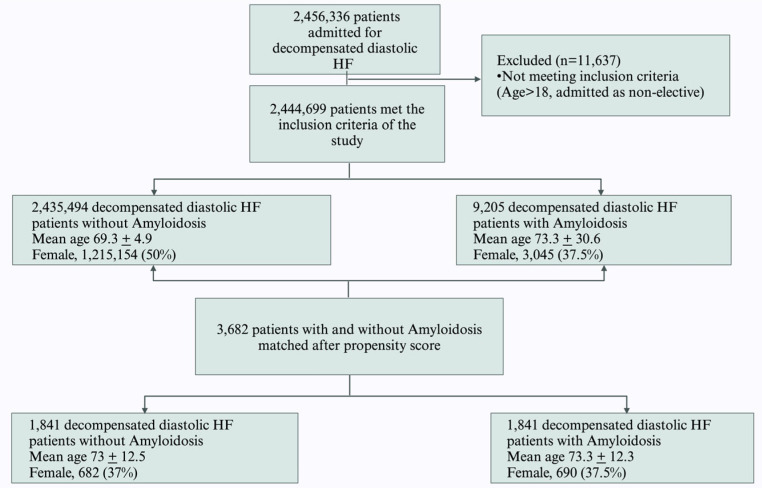
Flow diagram depicting selection of diastolic CHF patients for inclusion based on amyloidosis status and propensity-matched analysis (Created in BioRender. Dilli babu, A. (2025) https://BioRender.com/gtxh5cp, accessed on 3 May 2025).

The study enrolled patients admitted with a primary diagnosis of decompensated diastolic HF. Patients were identified using the International Classification of Diseases-10 Clinical Modification [ICD-10-CM], primarily as I5030-I5033 and I5040-I5043. Patients with amyloidosis in the secondary-diagnosis fields were captured using codes E853, E854, and E858. We aimed to retrieve highly relevant results for both amyloidosis and diastolic CHF by using only ICD-10-CM codes strongly linked to these conditions. However, as there is no ICD-10-CM code specific to cardiac amyloidosis, we included all subtypes (ATTRh, ATTRwt, and AL) [9,10,11]. Additional ICD-10-CM codes used in the study are provided in the Supplementary Material. Patients meeting any of the following criteria were excluded: elective admission, age under 18 years, transferred to or from another facility, and missing information. The remaining patients were divided into two study groups based on the presence of amyloidosis. The selection criteria and procedure for propensity-matched analysis of decompensated diastolic HF patients based on amyloidosis status are illustrated in Figure 1.

### 2.2. Study Variables

The primary outcome was in-hospital mortality. Secondary outcomes were rates of acute myocardial injury (AMI), cardiogenic shock, cardiac arrest, tracheal intubation, mechanical ventilation, acute kidney injury (AKI), ventricular tachycardia (VT), and ventricular fibrillation (VF), as well as length of stay (LOS) and total hospitalization charges.

### 2.3. Statistical Analysis

Continuous variables were presented as weighted means ± standard deviation (SD) and compared using Student’s *t*-test, while categorical variables were reported as numbers and percentages and compared using the Chi-square test. Following matching, univariate and multivariate analyses were conducted to compare the amyloidosis-present and amyloidosis-absent cohorts. Independent variables with *p*-values < 0.05 in univariate analyses were used to construct a multivariate regression model for the matched cohort, with primary endpoints as the dependent variable. All *p*-values were two-sided, with statistical significance set at 0.05 (Figure 1). Statistical analyses were conducted using STATA statistical software Version 18 [StataCorp. 2023. Stata 18 Base Reference Manual. College Station, TX, USA: Stata Press] [12].

## 3. Results

### 3.1. Baseline Patient Characteristics Before and After Propensity Matching

Prior to propensity matching, in an analysis of 2,444,699 patients hospitalized for decompensated diastolic HF, when compared to patients with a secondary diagnosis of amyloidosis, patients without a secondary diagnosis of amyloidosis showed a higher burden of comorbidities, as measured by the Charlson comorbidity index (CCI). Specifically, the patient population with secondary diagnosis of amyloidosis were less likely to have histories of diabetes mellitus (DM), hypertension (HTN), coronary artery disease (CAD), peripheral artery disease (PAD), history of myocardial infarction (MI) and percutaneous coronary intervention (PCI), smoking, and obesity; however, chronic kidney disease (CKD) and atrial fibrillation (AF) were more prevalent among patients with diastolic heart failure (HF) and amyloidosis (Table 1).

After 1:1 propensity matching, a total of 3682 patients were identified and divided into two cohorts, each comprising 1841 patients, based on the presence or absence of amyloidosis in diastolic HF with comparable baseline characteristics. Patients with diastolic HF and amyloidosis tended to be male, older, and of African American ethnicity, with a higher prevalence of CKD and atrial fibrillation (*p* < 0.05). However, the prevalence of major cardiovascular risk factors like HTN, DM, smoking, obesity, and COPD were similar between diastolic HF patients with and without amyloidosis. Geographically, the northeastern United States had the highest prevalence of amyloidosis and a higher likelihood of treatment at teaching hospitals (Table 2).

### 3.2. Inpatient Outcomes upon Propensity Match

Patients with decompensated diastolic heart failure (HF) and concurrent amyloidosis had longer hospital stays (7.1 days vs. 5.7 days, *p* < 0.05) and higher total hospital charges ($85,594 vs. $48,484, *p* < 0.05) on multivariate analysis. In addition, these patients had higher inpatient mortality rates compared to those without a history of amyloidosis (4.0% vs. 2.7%, aOR 1.48, 95% CI 1.22–2.13, *p* = 0.03) and exhibited an increased prevalence of acute myocardial injury (AMI), cardiogenic shock, and ventricular tachycardia (VT) (Table 3).

## 4. Discussion

**Figure 2:**
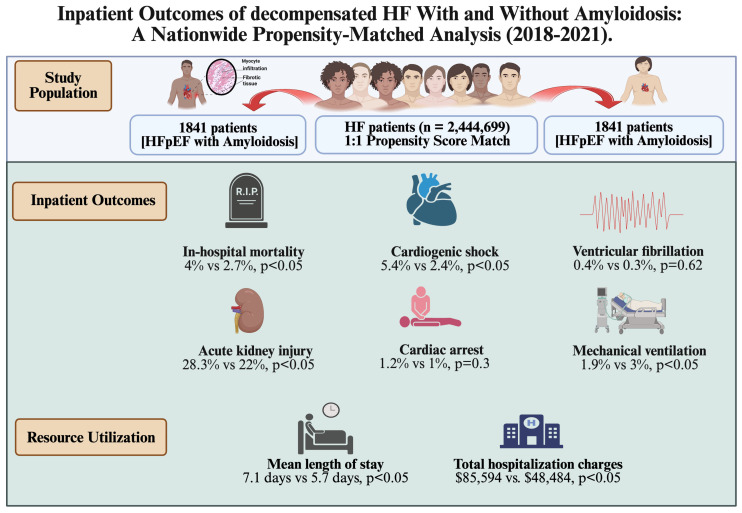
Central illustration. (Created in BioRender. Dilli babu, A. (2025) https://BioRender.com/x21b877, accessed on 3 May 2025).

Cardiac involvement is common in amyloidosis and often results in diastolic heart failure and reduced life expectancy. This study analyzed 2.4 million patients with decompensated diastolic HF treated between 2018 and 2021. The propensity-matched cohort analysis revealed significant findings: decompensated diastolic HF with amyloidosis was associated with higher rates of inpatient mortality, cardiogenic shock, acute myocardial injury (AMI), ventricular tachycardia (VT), need for mechanical ventilation, and acute kidney injury (AKI), as well as longer hospital stays and increased total hospitalization charges compared to decompensated diastolic HF without amyloidosis. However, differences in the likelihood of in-hospital cardiac arrest and rates of ventricular fibrillation were not statistically significant between the two groups. (Figure 2, central illustration).

Following propensity-matched analysis, decompensated diastolic HF patients with amyloidosis were primarily males aged over 70 and of African American ethnicity [5] and had a higher likelihood of inpatient mortality, cardiogenic shock, and acute myocardial injury. Interestingly, patients with diastolic HF with amyloidosis had a longer mean length of stay (7.1 vs. 5.7 days) and higher hospitalization charges ($85,594 vs. $48,484). This increased healthcare utilization could be attributed primarily to the irreversible nature of the disease and its poor prognosis, especially for AL amyloidosis [9,13]. A recent study by Gilstrap et al. found that although the overall prevalence of amyloidosis with heart failure (HF) is low, the burden of coexisting amyloidosis among hospitalized HF patients who are Medicare beneficiaries is on the rise [10]. Similarly, other studies have reported a temporal increase in the diagnosis of HF among patients with amyloidosis, especially in males of African American ethnicity aged over 70 [11,14].

This increasing recognition is partly driven by advancements in cardiac imaging modalities, such as speckle-tracking echocardiography (STE), which enable the comprehensive evaluation of indices of left atrial (LA) strain [15] and myocardial work (MW) [16]. LA-strain analysis using STE has emerged as a valuable tool not only for differentiating cardiac amyloidosis (CA) from phenotypically similar conditions such as hypertrophic cardiomyopathy (HCM), but also for identifying individuals at increased risk for arrhythmias and for assessing the extent of cardiac involvement and therapeutic response. In addition, LA-strain parameters have shown significant correlations with key echocardiographic markers, including left ventricular (LV) mass index, LA volume index, E/e′ ratio, and LV global longitudinal strain, and they have been independently associated with atrial fibrillation [15]. Concurrently, significant impairments in the global work index (GWI) and global constructive work (GCW) have been observed in patients with transthyretin cardiac amyloidosis (ATTR), underscoring the value of MW indices for identifying functional myocardial abnormalities in this population [16].

Clinical complexities in managing diastolic HF [17] in individuals are mainly attributed to the cytotoxicity associated with amyloid infiltration, which leads to ventricular dysfunction [13] and results in cardiac failure that is largely resistant to many common heart-failure therapies [18]. The escalating incidence of hospital admissions among amyloidosis patients underscores the necessity of scrutinizing these hospitalizations [19,20]. Such scrutiny holds promise for delineating avenues for cost mitigation and addressing unmet healthcare demands within this demographic; it thus should receive substantial attention in forthcoming scholarly investigations.

In findings consistent with those of prior studies, we observed a lower prevalence of chronic pulmonary disease and risk factors for traditional heart failure (HF), including hypertension, diabetes mellitus, coronary artery disease, and obesity, in patients with HF and amyloidosis [9,14]. These findings indicate that the progression and development of diastolic HF in patients with amyloidosis may not be entirely driven by pathways mediated by traditional risk factors. However, patients with diastolic HF with amyloidosis exhibited a higher prevalence of chronic kidney disease. Considering that amyloidosis is a systemic disease, these findings are not surprising; amyloid deposition may lead to CKD [21]. Further studies are needed to elucidate the causal role of amyloidosis in the pathogenesis of diastolic HF.

The study revealed that individuals with decompensated diastolic HF and amyloidosis had higher odds of experiencing ventricular tachycardia (VT) and atrial fibrillation (AF), which is a finding consistent with prior research [22]. While ventricular fibrillation (VF) can occur, the primary cause of death is likely electromechanical dissociation leading to pulseless electrical activity (PEA) [23]. Atrial arrhythmias seem to be more prevalent in patients with amyloidosis, with reports indicating an AF prevalence ranging from 69% to 88% [24]. While AF with amyloidosis does not impact all-cause mortality [23,25], it is strongly associated with incident and prevalent heart failure [26] and increases the risk of stroke [23]. Of note, there is a high prevalence of intracardiac thrombus even without AF; this condition warrants anticoagulation treatment irrespective of the CHADSVASC score [5,23,27].

A study investigating the placement of implantable cardioverter–defibrillators (ICDs) in patients assessed rates of ventricular arrhythmias (VAs) and ICD utilization and their impact on survival, revealing no survival benefits in patients with an ejection fraction (EF) < 35% [23,28]. Although the Heart Rhythm Society (HRS) guidelines assign a Class IIb indication to ICD placement in patients with AL cardiac amyloidosis (AL-CM) and non-sustained ventricular tachycardia, with an expected survival >1 year, the role of ICDs for primary prevention of sudden cardiac death in patients with ATTR-CM remains uncertain [23,28,29].

In this large multistate sample of hospitalizations for diastolic heart failure across the United States, the prevalence of diastolic HF with amyloidosis was 0.3%. Prior data have suggested that up to 13% of HF patients have an underlying diagnosis of amyloidosis [3]. A recent literature review revealed that 34–57% of patients with transthyretin amyloid cardiomyopathy (ATTR-CA) receive misdiagnoses such as hypertensive heart disease, hypertrophic cardiomyopathy, and ischemic heart disease [30]. Furthermore, consistent with findings from other research [10], our study revealed a higher prevalence of hospitalizations for decompensated diastolic HF with amyloidosis in the northeastern states, where patients were primarily managed in large urban hospitals. Conversely, most southern and western states, where a majority of these patients were African American (AA), had lower rates of hospitalization [31]. Despite amyloidosis being prevalent in the general population, the low prevalence of diastolic HF among amyloidosis patients (0.3%), as observed in this study, suggests underdiagnosis and misdiagnosis. This issue is particularly significant given the disproportionate prevalence of transthyretin amyloidosis (ATTR) among African Americans [32]. These findings further support the notion that amyloid care may be fragmented, emphasizing the need for improved recognition of amyloid disease and coordination of care.

## 5. Limitations

Due to coding limitations, we could not differentiate among amyloidosis subtypes (e.g., ATTRh, ATTRwt, AL), and specific ICD-10-CM codes for amyloid cardiomyopathy are not available. Consequently, we lacked sensitivity and specificity data for the ICD-10 codes, which served as a limitation in diagnostic accuracy. Moreover, we lacked treatment details, lab values, data on treatment duration, and cause-of-death analysis, which hindered our ability to assess disease severity. Additionally, the absence of post-discharge and follow-up data limited assessment of long-term outcomes. Our cross-sectional data allowed for the establishment only of association, not causation. Recognition of the limitations of retrospective registry data, including potential selection bias and reliance on ICD codes, is crucial. Despite limitations, utilizing the extensive and validated NIS database was pivotal in understanding real-world disease prevalence and outcomes, guiding the formation of future research hypotheses about patients with heart failure and amyloidosis.

## 6. Conclusions

In our analysis, we compared hospitalized patients with decompensated diastolic HF with and without a secondary diagnosis of amyloidosis using data from a national U.S. database. The subgroup with diastolic HF and amyloidosis displayed significantly higher risks of adverse outcomes, including inpatient mortality, cardiogenic shock, acute myocardial infarction (AMI), ventricular tachycardia (VT), mechanical ventilation, and acute kidney injury (AKI), compared to those without amyloidosis. Interestingly, patients with decompensated diastolic HF with amyloidosis had a longer mean length of stay and higher mean hospitalization charges. Additionally, despite only 0.3% of patients being identified as having diastolic CHF and amyloidosis, we noted a disproportionate prevalence of transthyretin amyloidosis (ATTR) within the African American population. Given the increasing recognition of diastolic HF with amyloidosis and the availability of novel therapies that can increase life expectancy, it is crucial to implement multicomponent screening protocols to enable early detection of amyloidosis. This effort may also help identify areas for cost-saving measures and warrant further research attention.

## Figures and Tables

**Table 1:** Patient characteristics before propensity matching.

Demographics	Diastolic HF Without Amyloidosis	Diastolic HF with Amyloidosis	*p*-Value
N = 2,444,699	N = 2,435,494	N = 9205	
Women, no. (%)	1,215,154 (50)	3450 (37.5)	<0.05
Age, mean, (SD)	69.3 (4.9)	73.3 (30.6)	<0.05
**Race/ethnicity, no. (%)**			
Caucasian	1,516,214 (63.4)	5045 (56.3)	
African American	519,705 (21.7)	2835 (31.6)	
Hispanic	228,145 (9.5)	635 (7)	
Asian or Pacific Islander	54,450 (2.3)	160 (1.8)	
Native American	14,490 (0.6)	20 (0.2)	<0.05
Other	56,450 (2.4)	265 (3)	
**Charlson comorbidity index score, no. (%)**			
1	354,350 (14.5)	1365 (14.8)	
2	627,065 (25.7)	1770 (19.2)	
3	565,580 (23.2)	2165 (23.5)	
>4	888,500 (36.5)	3905 (42.4)	<0.05
**Median annual income in patients’ zip code, no. (%)**			
$1–45,999	833,005 (35)	2520 (27.8)	
$46,000–58,999	634,280 (26.5)	2065 (22.8)	
$59,000–78,999	525,490 (22)	1995 (22)	
>$79,000	396,140 (16.6)	2480 (27.4)	<0.05
**Insurance type, no. (%)**			
Medicare	1,672,154 (70.3)	7025 (77.6)	
Medicaid	324,430 (13.6)	590 (6.5)	
Private HMO	293,155 (12.3)	1305 (14.4)	
Self-pay	88,475 (3.7)	135 (1.5)	<0.05
**Hospital characteristics**			
**Hospital region, no. (%)**			
Northeast	442,375 (18.2)	2705 (29.4)	
Midwest	520,775 (21.4)	2255 (24.5)	
South	1,029,800 (42.3)	2705 (30)	<0.05
West	442,544 (18.2)	1495 (16.2)	
**Hospital bed size, no. (%)**			
Hospital teaching status	1,691,649 (69.5)	7790 (84.6)	
Small	587,219 (24)	1465 (16)	<0.05
Medium	731,864 (30)	2430 (26.4)	
Large	1,116,411 (45.8)	5310 (57.7)	
Urban	2,188,949 (90)	8830 (96)	<0.05
Rural	246,545 (10)	375 (4)	
**Medical comorbidities**			
Diabetes mellitus	743,450 (30.5)	1440 (15.6)	<0.05
Hypertension	2,257,474 (92.7)	7250 (78.8)	<0.05
Hyperlipidemia	1,210,130 (49.7)	4365 (47.4)	0.06
Obesity	673,780 (27.7)	1005 (11)	<0.05
Smoker	1,125,989 (46.2)	3220 (35)	<0.05
COPD	847,515 (34.8)	1755 (19)	<0.05
Chronic kidney disease	490,880 (20)	2800 (30)	<0.05
CLD	59,280 (2.4)	315 (3.4)	<0.01
Atrial fibrillation	901,860 (37)	4610 (50)	<0.05
Aortic stenosis	118,750 (4.9)	335 (3.6)	0.01
CAD	1,072,740 (44)	3315 (36)	<0.05
History of MI	333,410 (13.7)	950 (10.3)	<0.05
History of PCI	309,530 (12.7)	740 (8)	<0.05
PAD	97,420 (4)	260 (2.8)	0.01
Iron-deficiency anemia	166,960 (6.8)	685 (7.4)	0.33
ACD	367,830 (15)	1535 (17)	0.06
OSA	426,595 (17.5)	1465 (16)	0.08
Alcohol	77,185 (3.2)	50 (0.5)	<0.05

ACD, Anemia of chronic disease; CAD, coronary artery disease; COPD, chronic obstructive pulmonary disease; CLD, chronic liver disease; MI, myocardial infarction; OSA, obstructive sleep apnea; PCI, percutaneous coronary intervention; PAD, peripheral arterial disease.

**Table 2:** Patient characteristics after propensity matching.

Demographics	Diastolic HF Without Amyloidosis	Diastolic HF with Amyloidosis	*p*-Value
N = 3682	N = 1841	N = 1841	
Women, no. (%)	682 (37)	690 (37.5)	0.78
Age, mean, (SD)	73 (12.5)	73.3 (12.3)	<0.01
**Race/ethnicity, no. (%)**			
Caucasian	1346 (74.5)	1009 (56.3)	
African American	250 (13)	547 (29.6)	
Hispanic	106 (5.8)	127 (7)	
Asian or Pacific Islander	28 (1.5)	32 (1.8)	<0.05
Native American	24 (1.5)	24 (1.5)	
Other	52 (2.8)	53 (2.9)	
**Charlson comorbidity index score, no. (%)**			
1	356 (19.3)	273 (14.8)	
2	437 (23.7)	354 (19.2)	<0.05
3	396 (21.5)	433 (23.5)	
>4	652 (35.4)	781 (42.4)	
**Median annual income in patients’ zip code, no. (%)**			
$1–45,999	429 (23.5)	504 (27.8)	
$46,000–58,999	405 (22.2)	413 (22.8)	
$59,000–78,999	500 (27.5)	399 (22)	0.001
>$79,000	487 (26.7)	496 (27.4)	
**Insurance type, no. (%)**			
Medicare	1411 (78)	1405 (77.6)	
Medicaid	136 (7.5)	118 (6.5)	
Private HMO	224 (12.4)	261 (14.4)	0.09
Self-pay	39 (2)	27 (1.5)	
**Hospital characteristics**			
**Hospital region, no. (%)**			
Northeast	1334 (72.5)	541 (29.4)	
Midwest	233 (12.6)	451 (24.5)	
South	204 (11)	550 (29.8)	<0.05
West	70 (3.8)	299 (16.2)	
**Hospital bed size, no. (%)**			
Small	610 (33)	293 (16)	
Medium	594 (32.3)	486 (26.4)	<0.05
Large	637 (34.6)	1062 (57.7)	
Urban	1651 (89.7)	1766 (96)	<0.05
Rural	190 (10)	74(4)	
Hospital teaching status	1351 (73.4)	1558 (84.6)	<0.05
**Medical comorbidities**			
Diabetes mellitus	295 (16)	288 (15.6)	0.75
Hypertension	1453 (79)	1450 (78.7)	0.89
Hyperlipidemia	860 (46.7)	873 (47.4)	0.66
Obesity	200 (11)	201 (11)	0.76
Smoker	642 (35)	644 (35)	0.49
COPD	342 (18.6)	351 (19)	0.70
Chronic kidney disease	381 (20.7)	559 (30.4)	<0.01
CLD	57 (3)	63 (3.4)	0.57
Atrial fibrillation	503 (27.3)	922 (50)	<0.01
CAD	660 (35.8)	663 (36)	0.81
History of MI	171 (9.3)	190 (10.3)	0.29
History of PCI	144 (7.8)	148 (8)	0.80
PAD	58 (3)	52 (2.8)	0.56
Iron-def anemia	122 (6.6)	137 (7.4)	0.33
ACD	235 (12.7)	307 (16.7)	0.001
OSA	243 (13.2)	293 (15.9)	0.02
Alcohol	57 (3)	11 (0.5)	<0.05

ACD, Anemia of chronic disease; CAD, coronary artery disease; COPD, chronic obstructive pulmonary disease; CLD, chronic liver disease; MI, myocardial infarction; OSA, obstructive sleep apnea; PCI, percutaneous coronary intervention; PAD, peripheral arterial disease.

**Table 3:** Univariate and multivariate analysis of inpatient outcomes of decompensated diastolic HF patients with and without amyloidosis upon PSM matching.

Inpatient Outcomes	Univariate OR (95% CI)	*p*-Value	Multivariate OR (95% CI)	*p*-Value
AKI	1.37 (1.20–1.62)	<0.05	1.45 (1.24–1.70)	<0.05
AMI	1.69 (1.32–2.17)	<0.05	1.71 (1.33–2.19)	<0.05
Cardiogenic shock	2.34 (1.63–3.36)	<0.05	2.41 (1.68–3.47)	<0.05
Cardiac arrest	1.09 (0.60–1.98)	0.76	1.11 (0.61–2.01)	0.73
Inpatient mortality	1.46 (1.20–2.11)	0.03	1.48 (1.22–2.13)	0.03
Mechanical ventilation	0.61 (0.40–0.94)	0.03	0.62 (0.40–0.96)	0.03
Tracheal intubation	0.81 (0.51–1.27)	0.36	0.81 (0.52–1.29)	0.36
VT	2.37 (1.86–3.02)	<0.05	2.39 (1.87–3.05)	<0.05
VF	1.33 (0.46–3.85)	0.59	1.30 (0.44–3.78)	0.62

AKI, acute kidney injury; AMI, acute myocardial injury; VT, ventricular tachycardia; VF, ventricular fibrillation.

## Data Availability

Data are contained within the article.

## Supplementary Materials

The following supporting information can be downloaded at: https://www.mdpi.com/article/10.3390/jcdd12050190/s1.

## Abbreviations

ATTR-CA	Transthyretin cardiac amyloidosis
AL-CA	Immunoglobulin light-chain cardiac amyloidosis
ATTRh-CA	Hereditary transthyretin cardiac amyloidosis
ATTRwt-CA	Wild-type transthyretin cardiac amyloidosis
AMI	Acute myocardial Injury
AKI	Acute kidney injury
CHF	Congestive heart failure
CCI	Charlson comorbidity index
CS	Cardiogenic shock
GDMT	Guideline-directed medical therapy
HFpEF	Heart failure with preserved ejection fraction
NSTEMI	Non-ST-elevation myocardial infarction
STEMI	ST elevation myocardial Infarction
NIS	National Inpatient Sample
LOS	Length of stay
VT	Ventricular tachycardia
VF	Ventricular fibrillation
