# Supplementary material for: Comparative Insights on Inpatient Outcomes in Diastolic Heart Failure with and Without Amyloidosis: A Nationwide Propensity-Matched Analysis
Source: J Cardiovasc Dev Dis. 2025 May 16;12(5):190. doi: 10.3390/jcdd12050190 (PMC12111973; doi:10.3390/jcdd12050190)
Supplement: Supplementary file 1 [file jcdd-12-00190-s001.zip › jcdd-3492666-supplementary.pdf]

Supplemental Material

ICD-10 diagnostic codes

**Diagnosis / Procedure**	**International Classification of Diseases 10 Clinical Modification codes**
Heart failure	I50, I501, I502, I5020, I5021, I5022, I5023, I5030, I5031, I5032, I5033, I504, I5040, I5041, I5042, I5043, I508, I5081, I50811, I50812, I50812, I50813, I50814, I5082, I5083, I5084, I509, I110, I132
Amyloidosis	E8582, E8581, E8589, E859, E853, E854
NSTEMI	I214
Atrial fibrillation	I480, I481, I482
Diabetes mellitus	E1010, E1011, E10618, E10620, E10621, E10622, E10628, E10630, E10638, E10641, E10649, E1065, E1069, E108, E109, E1100, E1101, E11618, E11620, E11621, E11622, E11628, E11630, E11638, E11641, E11649, E1165, E1169, E118, E119, E1300, E1301, E13618, E13620, E13621, E13622, E13628, E13630, E13638, E13641, E13649, E1365, E1369, E138, E139, E1310, E1311
Hypertension	I10, I110, I119, I129, I120, I130, I1310, I1311, I132
Hyperlipidemia	E782, E784, E785
Obesity	E6601, E6609, E661, E662, E668, E669
Smoking	Z87891, F17200, F17201, F17203, F17208, F17209, F17211, F17213, F17220, F17221, F17223, F17228, F17229, F17290, F17291, F17293, F17298, F17299, Z720, F17210, F17218, F17219, Z720, F17210, F17218, F17219
CAD	I2510, I25110, I25111, I25118, I25119
History of MI	I252
History of PCI	Z955, Z9861
PAD	I739
Multiple Myeloma	C9000, C9001, C9002

Leukemia	C9100, C9101, C902, C9110, C9111, C9112, C9130, C9131, C9132, C9140, C9141, C9142, C9150, C9151, C9152, C9160, C9161, C9162, C91A0, C91A1, C91A2, C91Z0, C91Z1, C91Z, C9190, C9191, C9192
AKI	N170, N171, N172, N178, N179
CKD	N181, N182, N183, N184, N185, N189
COPD	J44, J449, J441, J4489, J440, J431, J432, J438, J439, J41 J410, J411, J418, J42
Pulmonary hypertension	I270, I272
Iron deficiency anemia	D500, D509
Atrial fibrillation	I480, I4811, I4819, I4820, I482, I4891,I4892
Chronic Kidney Disease	N18, N182, N1830, N1831, N1832, N184, N185, N186, N189
Anemia of chronic disease	D630, D631, D638
Chronic liver disease	K740, K741, K742, K743, K744, K745, K7460, K7469, K769
VT	I472
VF	I4901
Cardiogenic shock	R570
Cardiac arrest	I462, I468, I469
